# The Efficacy of Angiotensin Receptor-Neprilysin Inhibitor Versus Angiotensin-Converting Enzyme Inhibitor or Angiotensin Receptor Blocker Post Myocardial Infarction: A Meta-Analysis

**DOI:** 10.7759/cureus.46547

**Published:** 2023-10-05

**Authors:** Sohny Kotak, Warda Hassan, Marium Mehmood, Umesh Kumar, FNU Sagreeka, FNU Karishma, Pirya Kumari, FNU Pirya, Javeria Saquib, Amna Iqbal, Anosh Aslam Khan, Giustino Varrassi, Mahima Khatri, Satesh Kumar

**Affiliations:** 1 Internal Medicine, Dow University of Health Sciences, Karachi, PAK; 2 Medicine and Surgery, Shaheed Mohtarma Benazir Bhutto Medical College, Karachi, PAK; 3 Medicine, Ghulam Muhammad Mahar Medical College, Sukkur, PAK; 4 Internal Medicine, Ghulam Muhammad Mahar Medical College, Khairpur, PAK; 5 Medicine, Peoples University of Medical and Health Sciences, Nawabshah, PAK; 6 Medicine, Dow University of Health Sciences, Civil Hospital Karachi, Karachi, PAK; 7 Internal Medicine, Monmouth Medical Center, Long Branch, USA; 8 Pain Medicine, Paolo Procacci Foundation, Rome, ITA; 9 Medicine and Surgery, Dow University of Health Sciences, Karachi, PAK

**Keywords:** angiotensin-converting enzyme inhibitors, meta-analysis, mi, myocardial infarction, neprilysin inhibitor

## Abstract

Acute myocardial infarction (MI) is one of the leading global healthcare emergencies, contributing to over three million global deaths. The purpose of this study is to investigate further the efficacy of sacubitril/valsartan over angiotensin-converting enzyme inhibitors (ACEIs) or angiotensin receptor blockers (ARBs) in reducing the risk of heart failure (HF) in post-MI patients and providing a clear evidence-based medicine guideline for future use.

An electronic database search was conducted on English databases. Eight articles were included, fulfilling our inclusion criteria, i.e., adult patients of ≥18 years with a recent diagnosis of acute MI. Pooled analysis was done using Review Manager version 5.4.1 (Cochrane Collaboration, London, England), and the data for each outcome were analyzed as dichotomous variables.

A total of eight clinical trials were included in the meta-analysis. Six studies analyzed the sacubitril/valsartan and ACEI combination. The pooled analysis reported a significant increase in the risk of hypotension (relative risk {RR}: 1.29 {1.18, 1.41}) in the sacubitril/valsartan compared to the ACEI alone group. In addition, a significant increase was observed in the left ventricle ejection fraction (LVEF) after using the sacubitril/valsartan combination compared to using ACEI alone (RR: 3.08 {2.68, 4.48}). Furthermore, no significant difference was observed between the groups in terms of mortality rate (RR: 0.86 {0.73, 1.02}), the risk of heart failure (RR: 0.62 {0.39, 1.00}), the frequency of recurrent MI (RR: 0.86 {0.27, 2.76}), and the mean difference of N-terminal pro-B-type natriuretic peptide (NT-proBNP) (weighted mean difference {WMD}: -174.36 {-414.18, 65.46}) between both the groups. However, the sacubitril/valsartan combination proved to be beneficial in significantly reducing the risk of major adverse cardiac events (MACE) (RR: 0.64 {0.48, 0.84}) and rehospitalizations (RR: 0.53 {0.39, 0.71}) as compared to ACEI post MI. Additionally, sacubitril/valsartan and ARB's combination was reported in two studies. This led to a significant decrease in NT-proBNP concentration (WMD: -71.91 {-138.43, -5.39}) post MI in the sacubitril/valsartan combination group compared to the ARB usage alone. However, no significant difference was observed in the improvement of LVEF (WMD: 0.88 {-5.11, 6.87}) between both groups.

Although the sacubitril/valsartan combination has no difference in mortality and outcomes compared to ACEI, there is evidence that using it proves to be more beneficial post MI compared to ACEI and ARB usage alone.

## Introduction and background

Acute myocardial infarction (MI) is a significant global healthcare crisis, resulting in over three million deaths worldwide, with more than one million of these fatalities occurring in the United States [1]. The mortality rate of acute myocardial infarction has significantly decreased due to timely interventions such as percutaneous coronary intervention (PCI). As a result, the mortality rates associated with cardiovascular disease have also been reduced [2]. Recent research has revealed a noteworthy phenomenon: despite the successful treatment of acute myocardial infarction (MI), the affected infarcted area may undergo unfavorable remodeling. This remodeling directly contributes to the emergence of long-term complications and subsequently increases mortality [3,4].

One commonly observed complication after myocardial infarction is heart failure (HF), a significant predictor of mortality. The increasing incidence of heart failure following myocardial infarction has imposed a significant burden on healthcare systems globally. In recent decades, heart failure (HF) has been acknowledged as a progressive medical condition in which cardiac remodeling plays a crucial role in its initiation. The activation of multiple neuroendocrine systems significantly influences the remodeling process. The sympathetic nervous system (SNS), renin-angiotensin-aldosterone system (RAAS), and natriuretic peptide system (NPS) are recognized as the critical neuroendocrine systems involved in the pathophysiology of heart failure. These systems can have beneficial and detrimental effects [3-5].

Angiotensin-converting enzyme inhibitors (ACEIs) have traditionally been considered the primary treatment for reducing mortality after acute myocardial infarction (MI), preventing the development of heart failure, and potentially preventing subsequent MIs [5]. Multiple clinical trials have conclusively shown the effectiveness of ACE inhibitors in reducing the neurohormonal impact of the renin-angiotensin-aldosterone system (RAAS) on cardiac remodeling and preventing further decline in cardiac function [6]. In 2015, a significant advancement occurred with the approval of sacubitril/valsartan, the pioneering angiotensin receptor-neprilysin inhibitor (ARNI). This development has had a transformative impact on the field of cardioprotective medicine. This novel category of medications possesses a distinctive capability to obstruct the angiotensin II receptor while simultaneously suppressing the neprilysin enzyme, exerting an effect on the renin-angiotensin-aldosterone system (RAAS) and the natriuretic peptide system (NPS), correspondingly. The superior efficacy of ARNI compared to ACEI has been demonstrated in the Prospective Comparison of ARNI with ACEI to Determine Impact on Global Mortality and Morbidity in Heart Failure (PARADIGM-HF) trial, where the combined inhibition of both the RAAS and NPS resulted in significant reductions in mortality and morbidity [7].

In this meta-analysis, a thorough search and systematic evaluation of the current literature was conducted to examine the comparative effectiveness of ARNI versus ACEI in mitigating the risk of heart failure after acute myocardial infarction. Our objective is to develop a comprehensive and evidence-based medical guideline for future clinical application, focusing on elucidating ARNI's potential advantages in this vulnerable patient population.

## Review

Methods

Search Strategy

A systematic literature search was conducted up until May 30, 2023, on the PubMed and Cochrane Central Register of Controlled Trials (CENTRAL) databases with the following subject keywords and their Medical Subject Heading (MeSH) terms: (sacubitril valsartan) OR (neprilysin inhibitor) AND (angiotensin receptor inhibitor) AND (angiotensin converting enzyme inhibitor) AND (post myocardial infarction). Google Scholar and Clinicaltrials.gov were searched for any studies that had not yet been published but had reported their results online. There was no language barrier, as all the studies retrieved in the search were in the English language. Two reviewers independently screened the search results. A third reviewer was consulted in case of discrepancies. Duplicates were removed, and studies were initially shortlisted based on title and abstract, after which the full text was assessed for eligibility. The references of the selected studies were also reviewed thoroughly to prevent any risk of selection bias.

Data Extraction

Two independent reviewers performed article identification and screened for titles and abstracts for inclusion in all potential studies. The results were retrieved and reviewed by a third investigator. Any possible inconsistencies within the lists were resolved through discussion and if needed by the third-party investigator. We retrieved full-text manuscripts of the initial inclusion criteria, and the same two independent reviewers screened them thoroughly. The reviewers only included the studies that satisfied the entire inclusion criteria, and for the articles that were excluded, a valid reason was recorded. We identified and excluded all duplicate articles for our final list.

Inclusion and Exclusion Criteria

**Figure 1:**
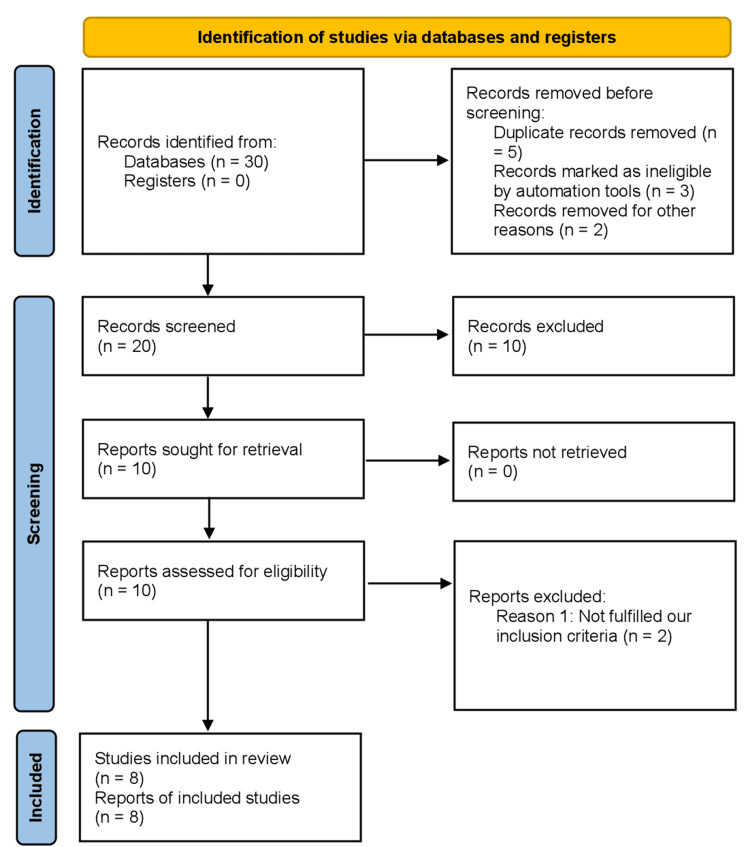
PRISMA flow chart for the included studies PRISMA: Preferred Reporting Items for Systematic Reviews and Meta-Analyses

This study was designed according to the Preferred Reporting Items for Systematic Reviews and Meta-Analyses (PRISMA) guidelines, as shown in Figure 1 [7,8]. After a thorough assessment of full-text manuscripts, studies with suitable design and sufficient data were included in the analysis. We included mostly randomized controlled trials (RCTs) in our meta-analysis with adult patients of a minimum of 18 years of age and older with a recent diagnosis of acute MI who were prescribed sacubitril/valsartan and either ACEI or angiotensin receptor blocker (ARB). In addition to this, all those studies that prescribed any other control regimen apart from ACEI and ARB were excluded. The primary outcomes were mortality, major adverse cardiovascular outcomes, heart failure, recurrent myocardial infarction, and improvement in left ventricle ejection fraction (LVEF) and N-terminal pro-B-type natriuretic peptide (NT-proBNP) concentration post MI, while a number of rehospitalizations and hypotension were the secondary outcomes.

Statistical Analysis

All statistical analyses were performed using Review Manager version 5.4.1 (2020) (Cochrane Collaboration, London, England). The data for each outcome were analyzed as dichotomous data. All tests were two-sided, and statistical significance was based on the 95% confidence interval (CI). Sensitivity analyses to assess the robustness of the results were conducted using the Mantel-Haenszel statistical method with a random-effects analysis model. Heterogeneity across the RCTs was assessed using I^2^ metrics (I^2^ range from 0% to 100%, with an I^2^ value of 25%-50% considered low, 50%-75% considered moderate, and >75% considered high).

Quality Assessment of the Included RCTs

**Figure 2:**
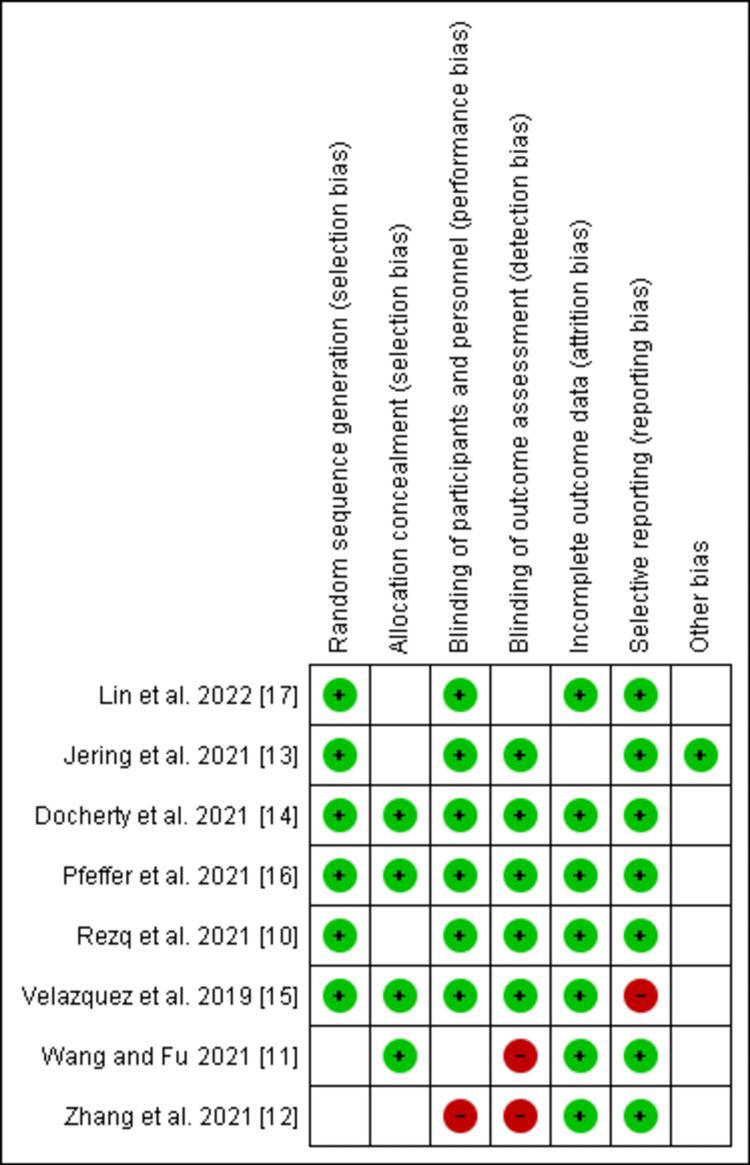
Cochrane risk-of-bias tool for the included RCTs Source: [10-17] RCTs: randomized controlled trials

The risk of bias for RCTs was calculated by the Cochrane risk-of-bias tool version 2 (RoB2) [9]. The tool included the following sections: random sequence generation, allocation concealment, blinding of participants, blinding of outcome assessment, incomplete outcome data, and selective reporting. Most of the studies were of fair to good quality according to their risk of bias, as shown in Figure 2.

Results

Study Characteristics

Our meta-analysis included eight prospective RCTs, which consisted of 7,318 participants in total, as shown in Figure 1. Table 1 lists the baseline characteristics for the control and intervention groups, and Table 2 provides a detail of outcomes from each study [10-17]. The included trials used varied doses of sacubitril/valsartan and the control group. Three thousand six hundred sixty-one patients were randomly assigned to the sacubitril/valsartan group throughout all the included studies, whereas 3,657 patients were randomly assigned to the control group. The mean age of the participants varied from 52-64 years between both groups across the included trials.

**Table 1:** Baseline characteristics of the included studies ARNI, angiotensin receptor-neprilysin inhibitor; SD, standard deviation; PPCI: primary percutaneous coronary intervention; N/A, not available; bid, twice daily; MTD, maximum tolerated dose; MI, myocardial infarction; AMI acute myocardial infarction

Study	Study design	Drugs			Total number of participants		Mean age ± SD		Male, N (%)		Diabetes mellitus, N (%)		History of hypertension, N (%)		Dyslipidemia, N (%)		Involvement of three coronary arteries, N (%)		Beta blockers at discharge, N (%)		Smoker, N (%)	
		Initial time	ARNI	Control	Control	ARNI	Control	ARNI	Control	ARNI	Control	ARNI	Control	ARNI	Control	ARNI	Control	ARNI	Control	ARNI	Control	ARNI
Rezq et al., 2021 [10]	Prospective, double-blinded, two-center, randomized study	After PPCI	Sacubitril/valsartan 100 mg, bid	Ramipril 5 mg, bid	100	100	57 ± 11.6	52 ± 9.2	88 (88)	86 (86)	34 (34)	40 (40)	38 (38)	34 (34)	94 (94)	86 (86)	12 (12)	8 (8)	N/A	N/A	74 (74)	66 (66)
Wang and Fu, 2021 [11]	Prospective, double-blinded, single-center, randomized study	After PPCI	Sacubitril/valsartan 100 mg, bid	Enalapril 5 mg, bid	69	68	60.56 ± 7.62	59.13 ± 7.15	54 (78.30)	52 (76.50)	20 (29.00)	15 (22.10)	28 (40.60)	32 (47.10)	39 (56.5)	34 (50.00)	10 (14.50)	8 (11.80)	43 (62.30)	45 (66.20)	39 (56.5)	34 (50.00)
Zhang et al., 2021 [12]	Prospective, controlled, single-center, randomized study	In 24 hours after PPCI	Sacubitril/valsartan, MTD	Perindopril, MTD	77	79	60.0 ± 10.9	60.3 ± 11.7	55 (71.4)	59 (74.7)	28 (35.4)	25 (31.6)	51 (66.2)	54 (68.4)	39 (50.6)	37 (46.8)	9 (11.7)	10 (12.7)	N/A	N/A	42 (54.5)	50 (63.3)
Jering et al., 2021 [13]	Prospective, double-blind, randomized, active-controlled trial	After seven days post MI	Sacubitril/valsartan 50 mg, 100 mg, and 200 mg	Ramipril 1.25 mg, 2.5 mg, and 5 mg	2,830	2,831	63.7 ± 11.5		4,302		2,400 (42.3)		3,672 (64.8)		2,959 (52.3)		N/A		N/A		1,199 (21.2)	
Docherty et al., 2021 [14]	Prospective, multicenter, randomized, double-blind, active-controlled trial	Three months after AMI	Sacubitril/valsartan 200 mg, bid	Valsartan 160 mg, bid	46	47	59.7 ± 10.1	61.8 ± 10.6	43 (93.5)	42 (89.4)	6 (13.0)	9 (19.1)	8 (17.4)	12 (25.5)	N/A	N/A	N/A	N/A	42 (91.3)	N/A	N/A	N/A
Velazquez et al., 2019 [15]	Prospective, multicenter, randomized, double-blind, active-controlled trial	24 hours to 10 days of acute decompensated heart failure	Sacubitril/valsartan 103/97, bid	Enalapril 10 mg, bid	441	440	63 (median)	61 (median)	308 (69.8)	327 (74.3)	N/A	N/A	N/A	N/A	N/A	N/A	N/A	N/A	263 (59.6)	45 (95.7)	N/A	N/A
Pfeffer et al., 2021 [16]	Prospective, multicenter, randomized, double-blind, active-controlled trial	0.5-7 days post MI	Sacubitril/valsartan 24/26, 49/51, and 103/97, bid	Ramipril 1.25, 2.5, or 5 mg, bid	2,831	2,830	63.5 ± 11.4	64.0 ± 11.6	2,131 (75.3)	2,167 (76.6)	1,180 (41.7)	1,221 (43.1)	1,831 (64.7)	1,845 (65.2)	N/A	N/A	N/A	N/A	2,414 (85.3)	262 (59.5)	N/A	N/A
Lin et al., 2022 [17]	Prospective, single-center, randomized trial	After PPCI	Sacubitril/valsartan 24/26 or 49/51, bid	Valsartan 40 mg or 80 mg, bid	54	55	59.74 ± 11.53	61.38 ± 12.31	47 (87.0)	49 (89.1)	12 (22.2)	15 (27.3)	20 (37.0)	23 (41.8)	N/A	N/A	N/A	N/A	N/A	2413 (85.2)	583 (20.6)	613 (21.7)

**Table 2:** Outcomes of the included studies ANRI, angiotensin receptor-neprilysin inhibitor; SD, standard deviation; MI, myocardial infarction; MACE, major adverse cardiovascular events; LVEF, left ventricle ejection fraction; HR, heart rate; N/A, not available; NT-proBNP, N-terminal pro-B-type natriuretic peptide

Study and year	Total number of participants	Number of patients in sacubitril/valsartan	Number of patients in controls	Death from any cause, n (%)		MI outcome, N (%)		Heart failure hospitalization, N (%)		MACE, N (%)		LVEF, mean ± SD		Adverse effects		HR mean (SD)		NT-proBNP mean (SD)		Rehospitalization, n (%)
				ANRI	Control	ANRI	Control	ANRI	Control	ANRI	Control	ANRI	Control	ANRI	Control	ANRI	Control	ANRI	Control	ANRI
Rezq et al., 2021 [10]	200	100	100	1	0	1 (1)	2 (2)	18 (18)	36 (36)	20 (20)	38 (38)	46.8 ± 12.5	42.09 ± 13.8	N/A	N/A	N/A	N/A	N/A	N/A	18 (18)
Wang and Fu, 2021 [11]	137	68	69	2 (2.90)	4 (5.80)	4 (5.90)	3 (4.30)	N/A	N/A	27 (39.70)	37 (53.60)	46.45 ± 5.32	43.65 ± 4.52	N/A	N/A	74.73 ± 8.77	77.30 ± 6.56	335.30 ± 73.29	593.24 ± 285.72	N/A
Zhang et al., 2021 [12]	156	79	77	N/A	N/A	N/A	-	3 (3.8)	10 (13.0)	3 (3.8)	7 (9.1)	46.1 ± 12.4	43.3 ± 11.7	N/A	N/A	N/A	N/A	1,760 ± 537	2,079 ± 615	5 (6.3)
Jering et al., 2021 [13]	5,669	2,831	2,830	N/A	N/A	N/A	N/A	N/A	N/A	N/A	N/A	N/A	N/A	N/A	N/A	N/A	N/A	N/A	N/A	N/A
Docherty et al., 2021 [14]	93	47	46	N/A	N/A	N/A	N/A	N/A	N/A	N/A	N/A	N/A	N/A	N/A	N/A	N/A	N/A	N/A	N/A	N/A
Velazquez et al., 2019 [15]	881	440	441	10 (2.3)	15 (3.4)	N/A	N/A	35 (8.0)	61 (13.8)	N/A	N/A	N/A	N/A	Hyperkalemia, hypotension, angiedema, and worsening renal function	N/A	N/A	N/A	35.2 (28.8-42.0)	-8.3 (-3.6 to -12.7)	35 (8.0)
Pfeffer et al., 2021 [16]	5,661	2,830	2,831	213 (7.5)	242 (8.5)	N/A	N/A	164/338 (48.5)	187/373 (50.1)	N/A	N/A	N/A	N/A	2,352 (83.1)	2,325 (82.1)	N/A	N/A	N/A	N/A	N/A
Lin et al., 2022 [17]	109	55	54	N/A	N/A	N/A	N/A	N/A	N/A	N/A	N/A	53.49 ± 6.46	49.58 ± 7.51	N/A	N/A	N/A	N/A	7,40.78 ± 1,156.24	2,720.8 ± 4,786	N/A

Outcomes

**Figure 3:**
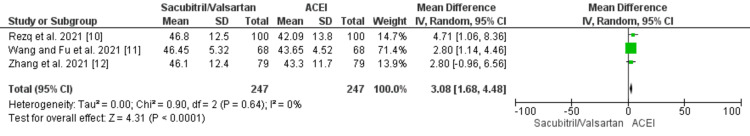
Forest plot of improvement in LVEF outcome for sacubitril/valsartan versus ACEI studies Source: [10-12] ACEI, angiotensin-converting enzyme inhibitor; SD, standard deviation; CI, confidence interval; IV, inverse variance; LVEF, left ventricle ejection fraction; df, degrees of freedom

Sacubitril/valsartan versus ACEI: This combination of intervention and control was analyzed by six studies. The pooled analysis of five studies reported a significant increase in the risk of hypotension (relative risk {RR}: 1.29 {1.18, 1.41}; p ≤ 0.00001) in the sacubitril/valsartan combination as compared to the ACEI alone group. In addition to this, when the improvement in the LVEF was compared between the intervention and control groups, a significant increase was observed after the usage of the sacubitril/valsartan combination as compared to using ACEI alone (RR: 3.08 {2.68, 4.48}; p < 0.00001) post MI as given in Figure 3.

**Figure 4:**
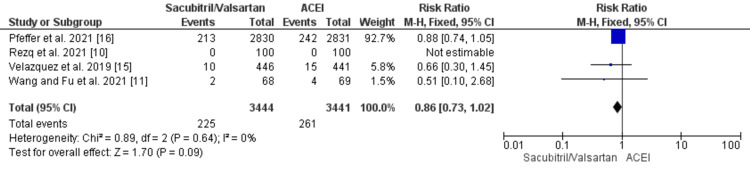
Forest plot of mortality outcome for sacubitril/valsartan versus ACEI studies Source: [10,11,15,16] ACEI, angiotensin-converting enzyme inhibitor; CI, confidence interval; df, degrees of freedom; M-H, Mantel-Haenszel

**Figure 5:**
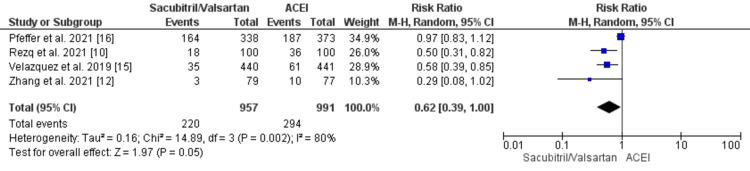
Forest plot of heart failure outcome for sacubitril/valsartan versus ACEI studies Source: [10,12,15,16] ACEI, angiotensin-converting enzyme inhibitor; CI, confidence interval; df, degrees of freedom; M-H, Mantel-Haenszel

Furthermore, no significant difference was observed between the groups in terms of an increase in the mortality rate (RR: 0.86 {0.73, 1.02}; p = 0.09) and the risk of heart failure (RR: 0.62 {0.39, 1.00}; p = 0.05) post MI, as shown in Figure 4 and Figure 5.

**Figure 6:**
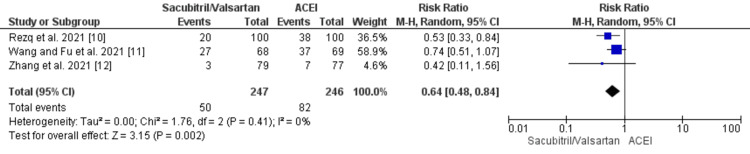
Forest plot of major adverse cardiac event (MACE) outcome for sacubitril/valsartan versus ACEI studies ACEI, angiotensin-converting enzyme inhibitor; CI, confidence interval; df, degrees of freedom; M-H, Mantel-Haenszel Source: [10-12]

However, the sacubitril/valsartan combination proved to be beneficial in significantly reducing the risk of major adverse cardiac events (MACE) (RR: 0.64 {0.48, 0.84}; p = 0.002) and rehospitalizations (RR: 0.53 {0.39, 0.71}; p < 0.00001) post MI, as shown in Figure 6.

**Figure 7:**
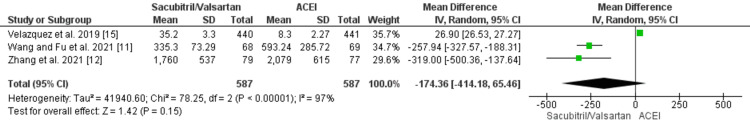
Forest plot of NT-proBNP outcome for sacubitril/valsartan versus ACEI studies Source: [11,12,15] ACEI, angiotensin-converting enzyme inhibitor; SD, standard deviation; CI, confidence interval; IV, inverse variance; df, degrees of freedom; NT-proBNP, N-terminal pro-B-type natriuretic peptide

Moreover, an insignificant difference was observed in terms of the frequency of recurrent MI (RR: 0.86 {0.27, 2.76}; p = 0.79) and the mean difference of NT-proBNP (RR: -174.36 {-414.18, 65.46}; p = 0.15) between both groups post MI, as shown in Figure 7.

**Figure 8:**
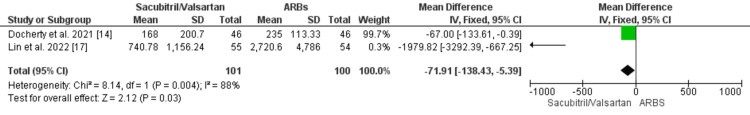
Forest plot for sacubitril/valsartan versus ARB N-terminal pro-B-type natriuretic peptide (NT-proBNP) outcome Source: [14,17] ARB, angiotensin receptor blocker; SD, standard deviation; CI, confidence interval; IV, inverse variance; df, degrees of freedom

Sacubitril/valsartan versus ARBs: A total of two studies analyzed this combination of intervention and control between sacubitril/valsartan and ARBs post MI. This led to a significant decrease in NT-proBNP concentration (weighted mean difference {WMD}: -71.91 {-138.43, -5.39}; p = 0.03) post MI in the sacubitril/valsartan combination group as compared to the ARB usage alone, as illustrated in Figure 8.

**Figure 9:**
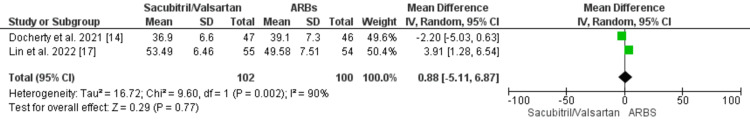
Forest plot for sacubitril/valsartan versus ARB left ventricle ejection fraction (LVEF) outcome Source: [14,17] ARB, angiotensin receptor blocker; SD, standard deviation; CI, confidence interval; IV, inverse variance; df, degrees of freedom

However, no significant difference was observed in the improvement of LVEF (WMD: 0.88 {-5.11, 6.87}; p = 0.77) between both groups, as given in Figure 9.

Discussion

In this extensive meta-analysis, our objective was to provide insights into the comparative outcomes of administering sacubitril/valsartan versus ACE inhibitors (ACEIs) or angiotensin receptor blockers (ARBs) to individuals who have suffered from acute myocardial infarction (MI). Our analysis provides valuable insights into the potential benefits of sacubitril/valsartan in enhancing post-myocardial infarction (MI) outcomes, specifically in relation to the left ventricle ejection fraction (LVEF), N-terminal (NT) pro-B-type natriuretic peptide (BNP) levels, rehospitalization rates, and major adverse cardiac events (MACE).

One of the significant findings from our meta-analysis is the notable improvement in the left ventricle ejection fraction (LVEF) observed in the experimental group that received sacubitril/valsartan, in comparison to the control group that received angiotensin-converting enzyme inhibitors (ACEIs) or angiotensin receptor blockers (ARBs). This discovery is consistent with prior research conducted by Zaid Iskandar and Lang [18], Lui et al. [19], Xiong et al. [20], and Zhang et al. [21], collectively supporting the effectiveness of sacubitril/valsartan in improving left ventricle function. These results align with the findings from the PARADIGM-HF trial [22].

Our study additionally illustrates a significant decrease in the levels of NT-proBNP in the experimental group compared to the control group. This finding aligns with previous research conducted by Lui et al. [19] and Xiong et al. [20]. The pathophysiology of heart failure entails an insufficient response characterized by the activation of the renin-angiotensin-aldosterone system (RAAS), resulting in adverse effects such as vasoconstriction, hypertension, elevated aldosterone levels, heightened sympathetic activity, and eventual cardiac remodeling [23]. Simultaneously, the natriuretic peptide system becomes activated, leading to increased levels of B-type natriuretic peptide (BNP) and N-terminal pro-B-type natriuretic peptide (NT-proBNP) during episodes of heart failure exacerbation. The enzyme neprilysin is known to have a significant role in the degradation of natriuretic peptides [23]. Sacubitril, a prodrug, functions as a neprilysin inhibitor upon activation, extending these peptides' advantageous effects [23]. BNP and NT-proBNP are essential biomarkers utilized in diagnosing and prognosticating heart failure. The research about natriuretic peptides as biomarkers for heart failure shows excellent potential [24]. This further emphasizes our finding of a significant decrease in the risk of heart failure within the experimental group.

The meta-analysis revealed a noteworthy decrease in rehospitalization rates among the experimental group receiving sacubitril/valsartan. Additionally, no significant heterogeneity was observed. The results presented are consistent with the research conducted by Xiong et al. [20] and Zhang et al. [21], wherein both studies observed a reduction in rehospitalization rates for heart failure after acute myocardial infarction. In addition, a recent meta-analysis conducted by Zhang et al. supports the beneficial use of sacubitril/valsartan in the treatment of heart failure. The study highlights a decrease in hospitalizations among patients with heart failure and midrange ejection fraction (HFmEF), as well as heart failure with preserved ejection fraction (HFpEF) [25].

The findings of our study indicate a significant decrease in major adverse cardiac events (MACE) among the experimental group. These results align with the consistent findings reported in previous studies conducted by Lui et al. [19], Xiong et al. [20], and Zhang et al. [21]. The decrease in major adverse cardiovascular events (MACE) can be attributed to the progressive enhancement of cardiac contractility and the inhibition of ventricular remodeling, facilitated by using sacubitril/valsartan. Furthermore, the previous EVALUATE-HF study revealed that the oral administration of sacubitril/valsartan significantly improved ventricular remodeling compared to the oral administration of enalapril [26-29].

Our study had some limitations that added up to the heterogeneity in outcomes such as heart failure and NT-proBNP. The main contributing factor can be different doses of sacubitril/valsartan in the intervention group in different studies along with the different timings of the introduction of the intervention in the participants. Some of the studies introduced the intervention after primary PCI, whereas some introduced it before PCI just after the MI along with patients having different comorbidities before MI in each study that could have overall led to heterogeneous results.

## Conclusions

In conclusion, our meta-analysis presents strong evidence supporting the efficacy of sacubitril/valsartan as a promising therapeutic approach for reducing major adverse cardiac events (MACE) and rehospitalizations and improving left ventricle ejection fraction (LVEF) in patients following acute myocardial infarction. In addition, the significant decrease in NT-proBNP levels observed in the experimental group highlights the potential of sacubitril/valsartan in reducing the risk of heart failure. These findings add to the increasing amount of evidence supporting the clinical usefulness of sacubitril/valsartan in managing patients after a myocardial infarction. They highlight the significance of timely and effective pharmacological interventions in promoting the recovery of cardiac function and improving patient prognosis. However, it is necessary to conduct additional research, including carefully planned clinical trials, to validate and build upon these encouraging findings. This will also help refine the treatment guidelines for patients who have experienced a myocardial infarction.
